# Direct Colorimetry of Imipenem Decomposition as a Novel Cost-Effective Method for Detecting Carbapenemase-Producing Enterobacteria

**DOI:** 10.1128/spectrum.00938-22

**Published:** 2022-07-19

**Authors:** Stathis D. Kotsakis, Anastasia Lambropoulou, Georgios Miliotis, Eva Tzelepi, Vivi Miriagou, Leonidas S. Tzouvelekis

**Affiliations:** a Laboratory of Bacteriology, Hellenic Pasteur Institute, Athens, Greece; Instituto Oswaldo Cruz

**Keywords:** CPE, carbapenemases, colorimetry, imipenem

## Abstract

In the absence of a molecule that would collectively inhibit both metallo-β-lactamases and serine-reactive carbapenemases, containment of their genes is the main weapon currently available for confronting carbapenem resistance in hospitals. Cost-effective methodologies rapidly detecting carbapenemase-producing enterobacteria (CPE) would facilitate such measures. Herein, a low-cost CPE detection method was developed that was based on the direct colorimetry of the yellow shift caused by the accumulation of diketopiperazines—products of the acid-catalyzed imipenem oligomerization—induced by carbapenemase action on dense solutions of imipenem/cilastatin. The reactions were studied by spectrophotometry in the visible spectrum using preparations of β-lactamases from the four molecular classes. The effects of various buffers on reaction mixtures containing the potent carbapenemases NDM-1 and NMC-A were monitored at 405 nm. Optimal conditions were used for the analysis of cell suspensions, and the assay was evaluated using 66 selected enterobacteria, including 50 CPE as well as 16 carbapenemase-negative strains overexpressing other β-lactamases. The development of the yellow color was specific for carbapenemase-containing enzyme preparations, and the maximum intensity was achieved in acidic or unbuffered conditions in the presence of zinc. When applied on bacterial cell suspensions, the assay could detect CPE with 98% sensitivity and 100% specificity, with results being comparable to those obtained with the Carba NP technique. Direct colorimetry of carbapenemase-induced imipenem decomposition required minimum reagents while exhibiting high accuracy in detecting CPE. Therefore, it should be considered for screening purposes after further clinical evaluation.

**IMPORTANCE** Currently, the spread of multidrug-resistant (MDR) carbapenemase-producing enterobacteria (CPE), mostly in the clinical setting, is among the most pressing public health problems worldwide. In order to effectively control CPE, use of reliable and affordable methods detecting carbapenemase genes or the respective β-lactamases is of vital importance. Herein, we developed a novel method, based on a previously undescribed phenomenon, that can detect CPE with few reagents by direct colorimetry of bacterial suspensions and imipenem/cilastatin mixtures.

## INTRODUCTION

The development of a single molecule that would collectively inhibit all carbapenemases is a difficult task due to their different reaction mechanisms zinc dependent, metallo-β-lactamases (ΜBL) or serine reactive, classes A and D. Although molecules with such properties are currently being tested (e.g., cyclic boronates [1]), none have entered the clinical practice, and the latest therapeutic β-lactam/β-lactamase inhibitor combinations, encompassing the diazabicyclooctane class of compounds (e.g., avibactam and relebactam), are only active against serine-reactive enzymes (2). Given that carbapenemase-producing enterobacteria (CPE) commonly express coresistances to other drugs of choice, early detection and confinement of their sources is currently the main way to confront outbreaks of the respective infections in health care settings (3).

A high number of CPE diagnostic techniques are currently available (reviewed in references 4, 5). Yet, only a handful are suitable for the screening purposes of an infection control approach. An efficient technique for CPE screening should be high throughput, sensitive and specific, cost-effective, and able to detect even unknown carbapenemases while also providing information on the reaction mechanism (MBL or serine reactive). The above criteria are simultaneously satisfied by methodologies that detect the carbapenemase activity in dense bacterial suspensions using color development (e.g., Rapidec Carba NP, Blue-Carba, beta-Carba, and MAST Carba PAcE [6–9]). Although the current colorimetric techniques are relatively low cost, the cumulative financial burden during a screening would still be high for a limited-budget setting.

Recently, during the development of a technique that detects the imipenem acidic hydrolysis product using an ion-sensitive field effect transistor (10), we observed that reaction mixtures containing CPE yielded a yellowish color that gradually became more intense—something that did not occur for the carbapenemase-negative strains even after prolonged incubation. This phenomenon most likely resulted from the pH drop during imipenem hydrolysis and was due to the complex oligomerization reactions taking place in dense solutions of the compound under acidic conditions yielding chromophoric diketopiperazines (11). Herein, we showed that under the experimental conditions of the techniques detecting carbapenemase production utilizing pH changes, a color shift would occur due to imipenem decomposition, even in the absence of an indicator and that this can be used as a cost-effective alternative method for CPE screening.

## RESULTS AND DISCUSSION

### Spectrophotometric analyses of β-lactamase–imipenem/cilastatin mixtures in the visible.

β-Lactam hydrolysis is accompanied by shifts in absorption in the UV spectrum due to the opening of the four-member ring. The fact that carbapenemase action on imipenem solutions in the absence of a pH buffer leads to absorbance changes in the visible region of the light spectrum, as the yellow color development indicated, prompted us to study the phenomenon through spectrophotometry.

**FIG 1:**
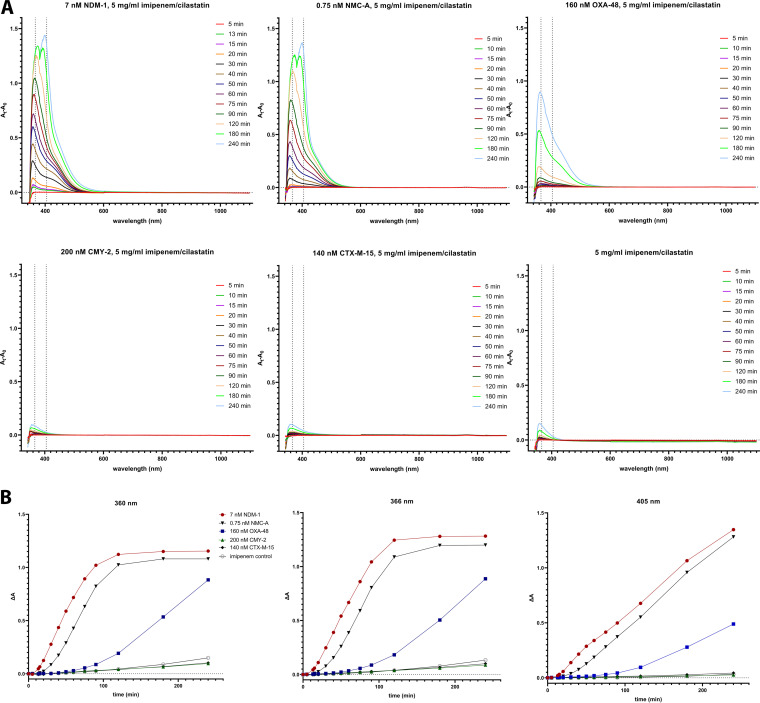
(A) Differential absorption spectra in the visible spectrum during incubation of various β-lactamases with 5 mg/mL imipenem/cilastatin. The efficient carbapenemases NDM-1 and NMC-A induced sharp absorbance shifts followed by the less potent OXA-48. Enzymes not exhibiting imipenemase activity caused marginal absorbance changes that were comparable to that of the control reaction. (B) Absorbance increases in three wavelengths corresponding to the detected peaks in differential absorption spectra. Monitoring the absorbance increase at 405 nm can clearly differentiate carbapenemases from noncarbapenemases.

The differential absorption spectra (Fig. 1A) in carbapenemase-containing reaction mixtures showed the accumulation of species that absorb in the violet region. The efficient carbapenemases NDM-1 and NMC-A assayed at nanomolar quantities induced shifts, which were apparent after 15 min. The less potent OXA-48 required longer reaction times and submicromolar quantities in order to observe the absorbance increases in the violet region (Fig. 1A, top). Conversely, CMY-2 and CTX-M-15—enzymes that do not exhibit meaningful imipenemase activity—yielded only minor absorbance increases after 3 h, similar to what was observed in the control reaction mixture containing solely imipenem/cilastatin (Fig. 1A, bottom). Therefore, the color shift was a phenomenon that was specifically observed for carbapenemases.

Multiple peaks were apparent with the λ_max_ initially being 360 nm and then increasing with time up to 370 nm. In the highly efficient NDM-1 and NMC-A, after 3 h of incubation, a second peak became predominant in the area of 400 nm while absorbance in the previous peak remained stable (Fig. 1A). The above data indicated that the reactions taking place during the action of carbapenemases resulted in the formation of more than one chromogenic product. By plotting the absorbance in various wavelengths, it was apparent that the carbapenemase activity could also be detected at 405 nm, although this required longer reaction times (Fig. 1B). Hence, the carbapenemase-induced color shift could be quantified in a clinical microbiology laboratory through widely available microplate absorbance readers instead of the UV-visible (UV-Vis) spectrophotometer used here.

### Effects of various solutions on carbapenemase-induced color formation.

**FIG 2:**
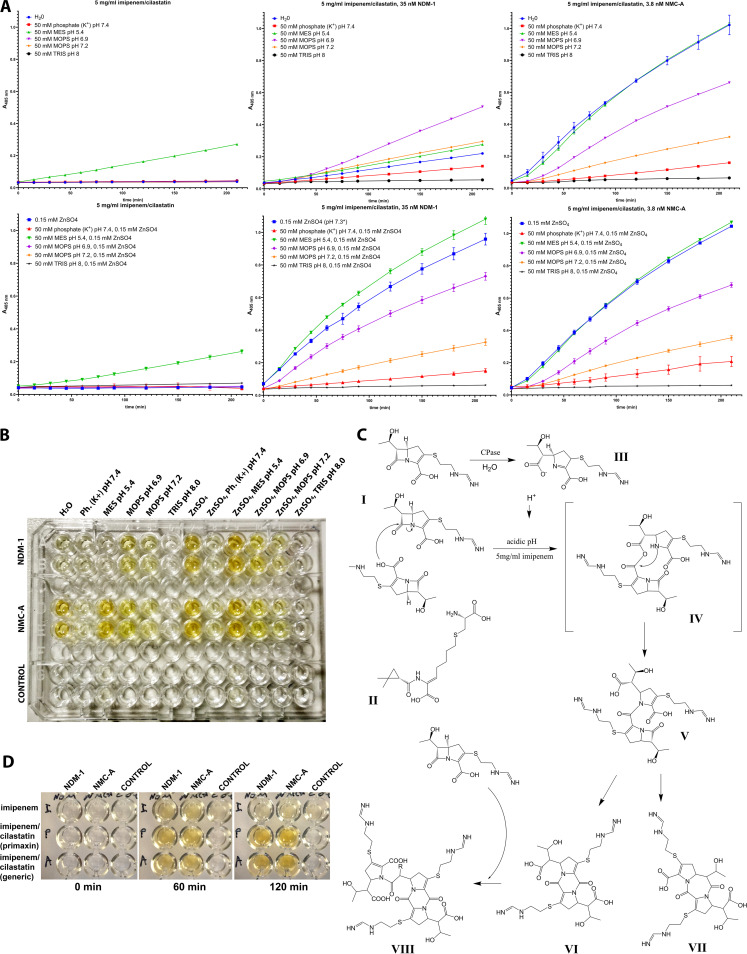
(A) Effects of various buffers on the color development induced by NDM-1 and NMC-A in the absence (top) and presence (bottom) of zinc cations estimated by absorbance measurements at 405 nm. In NDM-1-containing reactions (middle column) color development is significantly enhanced by zinc sulfate at a concentration of 0.15 mM, while the class A carbapenemase NMC-A (right column) yielded equivalent signals in both conditions. Color development was extended in acidic pH and in unbuffered conditions, with high alkalinity attenuating the reaction. In control reaction mixtures lacking a carbapenemase (left column), an absorbance increase was observed in the presence of 50 mM MES buffer, pH 5.4. (B) Color development in the above conditions as documented at the final point (*t* = 240 min). (C) A likely molecular mechanism for the carbapenemase induced yellow color. CPase, carbapenemase. (I) Imipenem; (II) cilastatin; (III) hydrolyzed imipenem. Compounds VI and VIII, containing a diketopiperazine ring, exhibit a λ_max_ at 360 nm and form yellow-colored solutions (11). (D) Effect of NDM-1 and NMC-A on pure imipenem and on the brand name imipenem/cilastatin formulation (Primaxin) in comparison with the generic imipenem/cilastatin formulation used in the study under the same conditions. Imipenem/cilastatin reaction mixtures yielded stronger and more stable yellow color compared to pure imipenem.

The effects of different buffers at various pH values in the presence and absence of zinc(II) on the occurrence of the yellowish color were assessed. In NDM-1-containing reaction mixtures, the color development was dependent on zinc, especially at the acidic pH of the 2-(*N*-morpholino)ethanesulfonic acid (MES) buffer as well as in unbuffered conditions, contrary to NMC-A and control experiments (Fig. 2A). Coloration induced by NMC-A was dependent on the pH and the buffering capacity of the solution. The highest absorbance increases were observed in reaction mixtures that did not contain a buffering salt (i.e., Η_2_Ο or 15 mM ZnSO_4_ reaction mixtures) and in the MES buffer at pH 5.4 (Fig. 2A and B, right graph column). In the presence of morpholinepropanesulfonic acid (MOPS), phosphate, and Tris buffers, the color development was significantly attenuated, as the alkalinity of the reaction environment increased (Fig. 2A and B). Similar observations were made for NDM-1 in zinc-supplemented solutions (Fig. 2A and B). In control reaction mixtures containing only imipenem, a moderate absorbance increase was observed in the acidic MES buffer irrespective of zinc ions with the remaining solutions being inert (Fig. 2A, left graph column). The specific requirement for Zn(II) in MBL reactions provided additional evidence that the phenomenon is indeed induced by the enzymatic hydrolysis of imipenem. It has been shown that low pH has a detrimental effect on MBL activity—probably due to the protonation of Asp120 of the second Zn(II) binding site that results in loss of one of the zinc ions—and that this can be countered by zinc supplementation of the reaction buffer (12, 13).

The dependence of the carbapenemase-induced color development on acidic pH or the absence of a buffering agent provided some evidence regarding the likely molecular bases of the phenomenon. It is known from stability studies of imipenem and the imipenem/cilastatin formulation that the compound decomposes in acidic pH at concentrations of ≥1 mg/mL through complex oligomerization reactions that lead to the formation of diketopiperazines yielding yellow-colored solutions (11, 14–17). Hence, a possible explanation for our data would be that as the enzymes hydrolyze the β-lactam ring of imipenem and the acidic hydrolysis product is accumulated, the pH is decreased, thus triggering secondary decomposition reactions leading to the formation of chromogenic diketopiperazines (Fig. 2C, compounds VI and VIII) (11).

The developed assay requires increased quantities of the substrate, and hence, to decrease the cost, we have used a commercially available generic imipenem/cilastatin formulation. In order to assert that the observed phenomenon is governed by the above mechanism, we assayed pure imipenem and the brand name imipenem/cilastatin formulation (Primaxin). After 60 min of incubation, the yellow color was developed in all reactions, with those of imipenem/cilastatin yielding stronger signals (Fig. 2D). At 2 h though, the color in the imipenem solution started to fade, indicating consumption of the chromophore product, in contrast to the imipenem/cilastatin solutions (Fig. 2D). The above results suggested that imipenem oligomerization caused by the acidification induced by the action of carbapenemases may indeed be the reason for the color development, at least in the initial reactions, with cilastatin having a yet unknown key role.

### Development of a CPE screening tool.

As assays with enzyme preparations indicated that the color development due to imipenem decomposition was specific for carbapenemases, we subsequently explored the use of this method as a diagnostic tool by testing cell suspensions of clinical isolates. Although the color shift was visually detectable, we quantified it through absorbance measurements at 405 nm using a microplate reader to improve objectivity.

**FIG 3:**
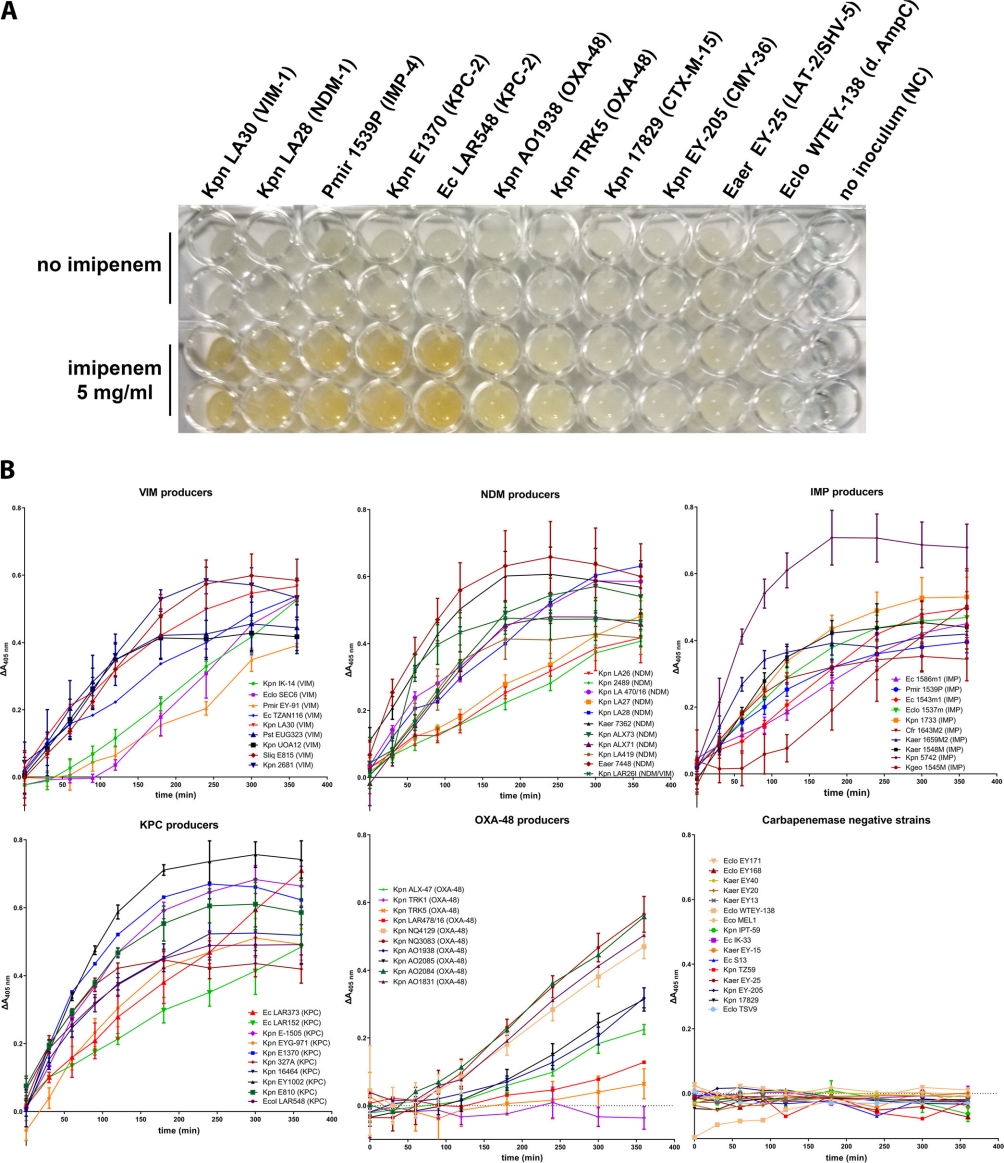
Application of the imipenem decomposition method on bacterial suspensions. (A) Color development at the endpoint (*t* = 360 min) of enterobacterial strains’ cell suspensions producing various types of carbapenemases and β-lactamases with no imipenemase activity. (B) Absorbance changes at 405 nm during the course of 6 h in imipenem/cilastatin-bacterial suspension mixtures of the strains assayed in the study. MBL- and KPC-producing strains yielded strong signals that could be detected in as early as 30 min. K. pneumoniae producing the OXA-48 class D carbapenemase yielded weaker responses, with one strain being identified as negative. The 16 non-carbapenemase producers did not cause any color shift even after 6 h of incubation.

The majority of the MBL-producing enterobacteria exhibited rapid color shifts that were also reflected on the measured absorbance (Fig. 3; Table 1). The weakest responses were observed with VIM-1-expressing strains, with three of them requiring more than 60 min of incubation for the yellow color to develop (Fig. 3). Nonetheless, all 30 ΜBL producers yielded high-intensity endpoint coloration with the maximum absorbance at 405 nm being in the range of 0.39 to 0.71 units (Fig. 3; Table 1). Fast color development was also evident for all KPC-2 class A carbapenemase producers tested with the maximum absorbance values ranging from 0.49 to 0.76 (Fig. 3; Table 1). Production of the less efficient OXA-48 class D carbapenemase required a longer incubation time for detection through the imipenem decomposition method with the yellow color developing after 90 to 120 min (Fig. 3). Furthermore, two strains isolated in the initial stages of the OXA-48 epidemic in the Near East yielded marginal or no color shifts (Klebsiella pneumoniae TRK-5 and TRK-1) (Fig. 3). These strains were found negative with the Carba NP method (Table 2). In the nine OXA-48-producing strains that yielded a response, the maximum absorbance varied between 0.06 and 0.57. The 16 isolates not producing a carbapenemase but overexpressing other β-lactamases did not yield any coloration even after 6 h of incubation (Fig. 3). The maximum absorbance values observed for these strains ranged from −0.01 to 0.03 units (Table 1). By applying threshold estimated trough receiver operating characteristic (ROC) analysis, the method could detect 30 out of 30 of the MBL strains within <30 to 180 min, 10/10 of KPC-2 producers in less than 30 to 60 min, and 9/10 of OXA-48 isolates in 90 to 360 min, while it excluded all of the non-carbapenemase producers as negatives (Table 1). Of note, two of the carbapenemase-negative isolates (K. pneumoniae EY-205 and 17829) gave false positive results when analyzed with the Carba NP technique (Table 2). The obtained data indicated that direct colorimetry could detect CPE with 98% sensitivity (1/50 false negatives) and 100% specificity (0/16 false positives).

**TABLE 1:** Clinical strains used in the study and absorbance changes at 405 nm during incubation of bacterial suspensions with 5 mg/mL imipenem/cilastatin^*a*^

Strain	β-lactamase(s)	Max Δ*Α*_405_	*t*_ΔΑ ≥ 0.05_ (min)	MIC (μg/mL)	
				Imipenem	Meropenem
VIM MBL producers					
Klebsiella pneumoniae IK14	VIM-1, CMY-2 type	0.53 ± 0.01	90	1	0.5
K. pneumoniae LA30	VIM-1, CMY-2 type	0.57 ± 0.01	<30	8	16
K. pneumoniae UOA12	VIM-1	0.42 ± 0.05	<30	8	2
K. pneumoniae 2681	VIM-27	0.58 ± 0.03	<30	8	32
E. coli TZ116	VIM-1, CMY-13	0.54 ± 0.01	<30	4	1
Enterobacter cloacae SEC6	VIM-1	0.53 ± 0.05	120–180	1	1
Proteus mirabilis EUG91	VIM-1, VEB-1	0.39 ± 0.01	120	4	≤0.125
Providencia stuartii EUG323	VIM-1	0.46 ± 0.08	<30	8	0.5
Serratia liquefaciens E815	VIM-1	0.59 ± 0.06	<30	32	16
NDM MBL producers					
K. pneumoniae LA26	NDM-1, CTX-M-15	0.42 ± 0.07	<30	16	32
K. pneumoniae LA27	NDM-1, CTX-M-15	0.48 ± 0.05	<30	8	8
K. pneumoniae LA28	NDM-1, CTX-M-15	0.63 ± 0.01	<30	8	64
K. pneumoniae 2489	NDM-1, CTX-M-15	0.41 ± 0.02	<30	32	64
K. pneumoniae LA470	NDM-1, CTX-M-15	0.59 ± 0.05	<30	8	64
K. pneumoniae LA419	NDM-1, CTX-M-15	0.43 ± 0.05	<30	8	32
K. pneumoniae ALX71	NDM-1, CTX-M-15	0.48 ± 0.02	<30	ND	8
K. pneumoniae ALX73	NDM-1, CTX-M-15	0.57 ± 0.07	<30	ND	8
K. pneumoniae LAR26I	NDM-1, VIM-1, CTX-M-15	0.48 ± 0.05	<30	16	64
Klebsiella aerogenes 7448	NDM-1	0.66 ± 0.10	<30	ND	8
K. aerogenes 7362	NDM-1	0.61 ± 0.07	<30	ND	8
IMP MBL producers					
K. pneumoniae 1733	IMP-4	0.53 ± 0.06	<30	ND	1
K. pneumoniae 5742	IMP-1	0.71 ± 0.08	<30	ND	8
K. aerogenes 1548m	IMP-4	0.45 ± 0.06	<30	ND	8
K. aerogenes 1659m2	IMP-4	0.42 ± 0.07	<30	ND	8
Kluyvera georgiana 1545m	IMP-4	0.50 ± 0.11	60–90	ND	1
E. coli 1543m1	IMP-4	0.49 ± 0.05	<30	ND	4
E. coli 1586m1	IMP-38	0.45 ± 0.05	<30	ND	0.5
E. cloacae 1537m	IMP-4	0.47 ± 0.03	<30	ND	8
P. mirabilis 1539p	IMP-4	0.39 ± 0.03	<30	ND	8
Citrobacter freundii 1643m2	IMP-38	0.35 ± 0.04	<30	ND	4
KPC-2 class A carbapenemase producers					
K. pneumoniae E971	KPC-2, SHV-5 type	0.49 ± 0.05	60	1	8
K. pneumoniae E1370	KPC-2	0.62 ± 0.01	<30	2	4
K. pneumoniae E1505	KPC-2	0.68 ± 0.04	<30	32	16
K. pneumoniae EY1002	KPC-2	0.76 ± 0.04	<30	ND	ND
K. pneumoniae 16464	KPC-2	0.53 ± 0.06	<30	ND	ND
K. pneumoniae 327A	KPC-2	0.44 ± 0.04	<30	ND	ND
K. pneumoniae E810	KPC-2	0.59 ± 0.09	<30	64	32
E. coli LAR548	KPC-2	0.49 ± 0.06	<30	ND	ND
E. coli LAR373	KPC-2	0.71 ± 0.03	<30	2	1
E. coli LAR152	KPC-2, CTX-M, OXA-1	0.49 ± 0.07	<30	2	2
OXA-48 class D carbapenemase producers					
K. pneumoniae ALX47	OXA-48, CTX-M-15	0.23 ± 0.02	180	4	8
K. pneumoniae LAR478	OXA-48, CTX-M-15	0.13 ± 0.01	240	8	8
K. pneumoniae AO2085	OXA-48, CTX-M-15	0.31 ± 0.03	180	ND	ND
K. pneumoniae AO1938	OXA-48, CTX-M-15	0.32 ± 0.03	180	ND	ND
K. pneumoniae AO1831	OXA-48, CTX-M-15	0.50 ± 0.07	90	ND	ND
K. pneumoniae AO2084	OXA-48, CTX-M-15	0.56 ± 0.01	90	ND	ND
K. pneumoniae NQ3083	OXA-48	0.57 ± 0.05	90	8	4
K. pneumoniae NQ4129	OXA-48	0.47 ± 0.03	90	4	4
K. pneumoniae TRK1	OXA-48, CTX-M-15	−0.04 ± 0.04	NA	8	32
K. pneumoniae TRK5	OXA-48, CTX-M-15	0.06 ± 0.04	360	16	32
Carbapenemase-negative strains					
K. pneumoniae TZ59	Species-specific SHV	−0.002 ± 0.003	NA	≤0.125	≤0.125
K. pneumoniae IPT59	GES-7, SHV-5	0.01 ± 0.03	NA	0.25	≤0.125
K. pneumoniae 17829	CTX-M-15, SHV-12	−0.01 ± 0.01	NA	16	16
K. pneumoniae EY-205	CMY-36, SHV-5	0.002 ± 0.03	NA	0.25	≤0.125
K. aerogenes EY-25	LAT-2+SHV-5	−0.01 ± 0.03	NA	16	8
K. aerogenes EY-13	Derepressed AmpC	−0.01 ± 0.04	NA	0.5	≤0.125
K. aerogenes EY-20′	GES, derepressed AmpC	0.001 ± 0.02	NA	2	2
K. aerogenes EY-40	SHV-5, derepressed AmpC	0.006 ± 0.07	NA	0.25	≤0.125
E. cloacae WTEY-138	Derepressed AmpC	0.009 ± 0.01	NA	0.5	≤0.125
E. cloacae WTEY-168	TEM-1, induc. AmpC	−0.01 ± 0.01	NA	0.5	≤0.125
E. cloacae WTEY-171	Derepressed AmpC	0.03 ± 0.01	NA	4	0.5
E. cloacae TSV9	GES-7	0.01 ± 0.03	NA	0.5	≤0.125
E. coli MEL1	LAT-3	0.02 ± 0.02	NA	0.5	≤0.125
E. coli IK33	CTX-M-15	0.003 ± 0.006	NA	≤0.125	≤0.125
E. coli S13	CTX-M-32	0.009 ± 0.005	NA	≤0.125	≤0.125
K. aerogenes EY-15	LAT-2	0.007 ± 0.004	NA	8	4

a ND, not determined; NA, not applicable; derepressed, derepressed expression of the chromosomal cephalosporinase; induc., inducible expression of the chromosomal cephalosporinase.

**TABLE 2:** Comparison of the direct colorimetry method with Rapidec Carba NP for selected strains

Strain	β-lactamase(s)	Direct colorimetry result	Carba NP result/color
K. pneumoniae LA30	VIM-1	Positive	Positive/yellow
E. coli TZ116	VIM-1, CMY-13	Positive	Positive/yellow
P. mirabilis EUG91	VIM-1, VEB-1	Positive	Positive/yellow
K. pneumoniae 2489	NDM-1/CTX-M	Positive	Positive/yellow
K. pneumoniae LA28	NDM-1/CTX-M	Positive	Positive/yellow
K. aerogenes 7362	NDM-1	Positive	Positive/yellow
K. pneumoniae E971	KPC-2, SHV-5 type	Positive	Positive/yellow
K. pneumoniae E1370	KPC-2	Positive	Positive/yellow
E. coli LAR548	KPC-2	Positive	Positive/yellow
K. pneumoniae ALX47	OXA-48, CTX-M-15	Positive	Positive/orange
K. pneumoniae LAR478	OXA-48, CTX-M-15	Positive	Positive/orange
K. pneumoniae TRK1	OXA-48, CTX-M-15	Negative	Negative/red
K. pneumoniae TRK5	OXA-48, CTX-M-15	Positive	Negative/red
K. pneumoniae EY-205	CMY-36/SHV-5	Negative	Positive/orange
K. pneumoniae 17829	CTX-M-15/SHV-12	Negative	Positive/orange
K. aerogenes EY-25	LAT-2/SHV-5	Negative	Negative/red
E. cloacae EY 138	Derepressed AmpC	Negative	Negative/red
E. coli IK33	CTX-M-15	Negative	Negative/red

**FIG 4:**
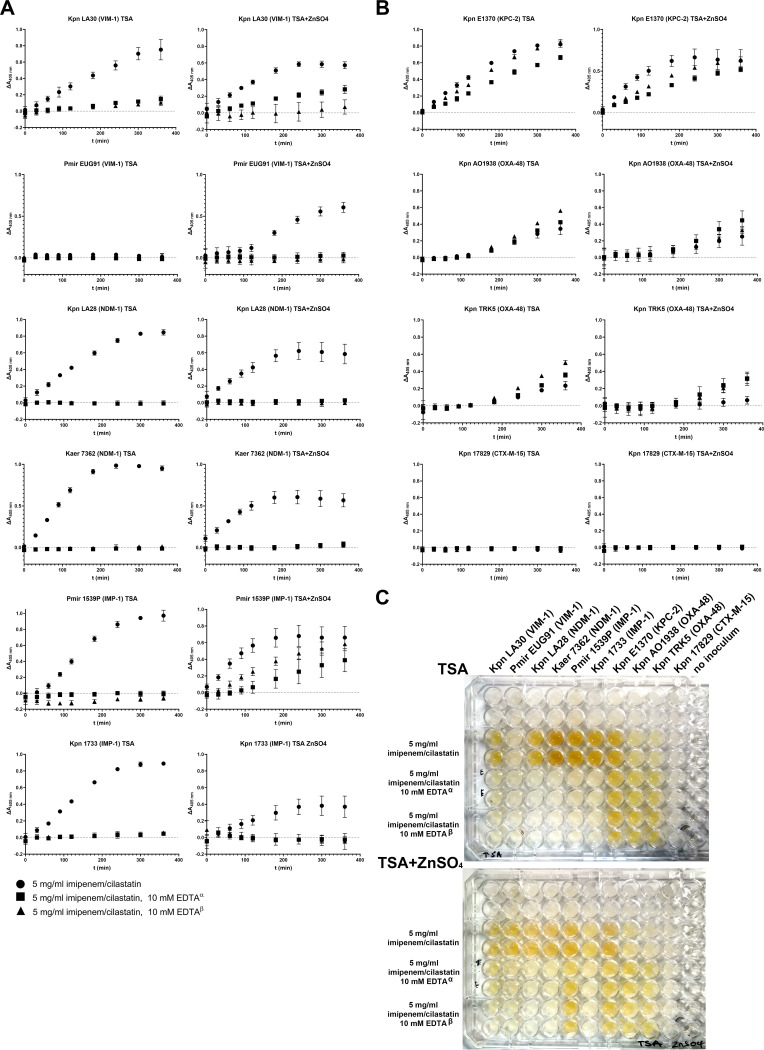
Differentiation of MBL and class A carbapenemase producers through the use of EDTA and effects of zinc cation supplementation of the growth medium. (A) Time courses of absorbance changes at 405 nm of MBL-producing strains in the presence and absence of 10 mM EDTA when bacteria were cultured without (left) and with zinc supplementation (right) in the TSA medium. Experiments were performed with EDTA added in the wells prior to reactants’ addition (black squares, α) or with bacterial cells suspended in a 20 mM EDTA solution (black triangles, β). EDTA inhibited the decomposition of imipenem in the majority of MBL strains when they were cultivated in the absence of zinc. P. mirabilis EUG91 did not yield any signal when grown on plain TSA. (B) Effects of EDTA and growth on zinc supplemented TSA on reactions containing strains producing serine-reactive β-lactamases. (C) Coloration observed in the above experiments after 6 h of incubation.

The ability of this method to discriminate between ΜBL and serine-reactive carbapenemase producers was assessed using EDTA as a chelating agent. Preliminary experiments were performed using 10 and 15 mM EDTA in the reaction mixtures. At these concentrations, the color formation observed in MBL-producing strains was attenuated, but a quenching in the coloration induced by KPC-2 producers was observed, probably due to an increase of the solution’s alkalinity (Fig. S1 in the supplemental material). Hence, 10 mM was selected as an optimal EDTA concentration. EDTA could efficiently inhibit the yellow color induced by suspensions of NDM-producing bacteria as well as of some VIM- and IMP-producing strains with insignificant effects on signals obtained from serine-reactive carbapenemase producers (Fig. 4). Color formation in the presence of EDTA was evident with a VIM-1-producing K. pneumoniae (Kpn LA30) and a Proteus mirabilis strain expressing the IMP-4 enzyme (Fig. 4A, right). Considering that the yellow color is formed indirectly through secondary reactions and not due to the direct action of carbapenemases, any residual imipenem hydrolysis may initiate the cascade leading to a positive result. In order to overcome this, we performed the same experiments with the bacterial suspensions being prepared in EDTA, which was then mixed with the imipenem/cilastatin solution. This modification permitted the inhibition of K. pneumoniae LA30 reactions but not those of the P. mirabilis IMP-4 strain that was still able to yield a strong coloration (Fig. 4A and C). The above may be due to the relative resistance of IMP enzymes to the action of EDTA combined with increased levels of the enzyme in the bacterium’s periplasm.

Indeed, as the strains were grown on zinc-supplemented media in order to assert high periplasmic levels of fully functional ΜBLs, the observed resistance of the color formation to EDTA inhibition may be due to increased enzyme quantities released in the solution. In order to assess this, we performed the same set of experiments with strains grown on plain tryptone soya agar (TSA). The results showed that without zinc supplementation, the color development could be inhibited by EDTA in the majority of the MBL-producing strains (Fig. 4A, left). However, P. mirabilis EUG91, which produces low quantities of VIM-1 (10), failed to give a positive reaction even after 6 h of incubation. Thus, EDTA inhibition could be used for the identification of MBL producers with the above method when bacteria are cultured without excess zinc, but this would reduce the sensitivity for some strains exhibiting low levels of functional periplasmic MBLs.

The color development induced by suspensions of serine-reactive CPE remained relatively unaffected by EDTA (Fig. 4B and C). It should be noted that in OXA-48 producers, the chelator increased the color intensity, probably through the release of more enzyme in the solution facilitated by its detrimental effect on cell wall integrity (Fig. 4B). Zinc supplementation of the growth medium seemed to affect the chromogenic reaction of positive strains yielding systematically lower endpoint absorbance increases even for the MBL-producing isolates (average difference of maximum absorbance increases between experiments utilizing plain and zinc supplemented TSA observed for each positive strain: ΔOD^TSA-max, TSA+ZnSO4-max^ = 0.26 ± 0.12; paired *t* test *P* < 0.001). Zinc ions are known to have a variety of effects on bacterial physiology (18), and therefore, we cannot yet provide an explanation for the above observation. Strains not producing a carbapenemase remained negative under all of the employed modifications of the method.

Based on these data, the use of direct colorimetry combined with various chelating agents in order to distinguish MBL from serine carbapenemase producers warrants further study.

### Conclusions.

Imipenem is the first clinically used carbapenem that exhibits increased stability to aminolysis compared to its natural counterpart thienamycin. Yet, the molecule still possesses an inherent instability in aqueous solutions compared to newer carbapenems (e.g., meropenem) (16, 19). Indeed, its decomposition in low pH results in complex degradation products not observed in the other members of the group (20). Herein, we showed that the accumulation of chromophoric decomposition products of imipenem in the acidic conditions induced by the action of carbapenemases can be used for the specific detection of CPE directly from bacterial suspensions. As the method was only evaluated against enterobacterial strains, it should not be used with other carbapenemase producers (e.g., nonfermenting Gram negative).

Direct colorimetry required minimum reagents, i.e., a solution of 10 mg/mL imipenem-10 mg/mL cilastatin containing 0.3 mM zinc sulfate, which can be prepared from any imipenem/cilastatin powder formulation for injection used in hospitals. The development of yellow color can be followed either through manual inspection or with a microplate reader capable of measuring absorbance at 405 nm, which would increase both throughput and objectivity. The cost of the method would be significantly lower (>100 fold) compared to that of the current commercial CPE detection colorimetric techniques, considering that a 500 mg imipenem/500 mg cilastatin vial (valued at €6.00, Greek market consumer prices [21]) would be sufficient for 500 reactions. Moreover, as direct colorimetry exhibited high accuracy in detecting CPE and discriminating non-carbapenemase producers, it fulfills the requirements of a successful CPE screening technique and merits further evaluation in a variety of clinical settings.

## MATERIALS AND METHODS

### β-Lactamase preparations.

Crude protein extracts containing β-lactamases were prepared from laboratory Escherichia coli clones replicating the recombinant plasmids pZE21-*bla*_NDM-1_ (E. coli C600Z1), pNTN3-*bla*_NMC-A_ (E. coli JM109), pZE21-*bla*_OXA-48_ (E. coli C600Z1), pBC-*bla*_CMY-2_ (E. coli DH5α), and pBC-*bla*_CTX-M-15_ (E. coli DH5α) overexpressing the NDM-1 (MBL), NMC-A (class A carbapenemase), OXA-48 (class D carbapenemase), CMY-2 (class C β-lactamase), and CTX-M-15 (class A β-lactamase–extended-spectrum β-lactamase [ESBL]) enzymes, respectively. In pZE21 clones, transcription of the cloned β-lactamase gene was induced by 200 ng/mL anhydrotetracycline, while in the remaining plasmids, expression was constitutively driven by natural promoters of the genes. Proteins were released through sonication (10) in 50 mM sodium phosphate buffer (pH 7) with the exception of the MBL preparations where a 50 mM HEPES, 50 μΜ ZnSO_4_ (pH 7.2) buffer was used. Hydrolysis of imipenem (NDM-1, NMC-A, and OXA-48), cephalothin (CMY-2), or cefotaxime (CTX-M-15) was measured by UV spectrophotometry. β-Lactamase concentration in the extracts was estimated using the initial velocities and the published steady state hydrolysis constants (22–26) by the Michaelis-Menten equation.

### Bacterial strains and susceptibility testing.

A total of 66 nonrepetitive enterobacterial strains were used in the study. These included 50 strains producing a carbapenemase and 16 strains producing β-lactamases with either marginal or no carbapenem hydrolytic activity (noncarbapenemases). The detailed β-lactamase content of each strain is given in Table 1. Isolates had been previously characterized using phenotypic and molecular techniques (27, 28). The uniqueness of strains belonging in the same species and exhibiting identical β-lactamase content was asserted through restriction fragment length polymorphism analysis using pulsed-field gel electrophoresis. The majority of strains had been isolated from clinical settings in Greece, save for nine IMP producers, which were of environmental origin (29).

Imipenem and meropenem MICs were determined using the microdilution method in Mueller-Hinton broth according to EUCAST recommendations. Carbapenems were tested at a concentration range from 0.125 to 128 μg/mL.

### Spectrophotometric analyses of imipenem decomposition in the visible.

In spectrophotometric analyses, a stock solution of 10 mg/mL imipenem-10 mg/mL cilastatin was used. It was prepared from a generic 500 + 500 mg imipenem/cilastatin powder for injection containing also 1.6 mmol of sodium bicarbonate (NaHCO_3_). Reconstitution was carried out using either a solution of 0.3 mM zinc sulfate (ZnSO_4_) or deionized water, and the resulting suspensions were stored as aliquots at −80°C until further use.

Acquisition of absorbance spectra was carried out using a Hitachi U-2001 UV-Vis double beam spectrophotometer in a quartz cuvette of 1 cm optical path. Each reaction had a volume of 1 mL and was prepared through 1:1 dilution of the imipenem/cilastatin-ZnSO_4_ stock solution in deionized water that resulted in the following composition: 5 mg/mL imipenem, 5 mg/mL cilastatin, 16 mM NaHCO_3_, and 0.15 mM ZnSO_4_ (pH 7.2 ± 0.1). Quantities of the β-lactamase preparations were added in the reaction mixture—with the buffering salt included in the crude protein extract having a final concentration of no more than 2 mM—and the spectrum from 342 nm to 1,100 nm was scanned at a rate of 800 nm · min^−1^ at various time intervals. Differential absorption spectra were obtained through subtraction of the initial spectrum from the spectra obtained at each time point. A control reaction lacking a β-lactamase was also performed as above.

The effects of zinc, pH, and various buffers on the carbapenemase-induced color development were examined using a Dynex MRX absorbance microplate reader. Readings were obtained at 405 nm with the reference filter being set at 630 nm. Here, the imipenem/cilastatin-water stock solution was used, which was diluted 1:1 in (i) deionized water, (ii) 0.1 M 2-(*N*-morpholino)ethanesulfonic acid (MES) (pH 5.4), (iii) 0.1 M 3-(*N*-morpholino)-propanesulfonic acid (MOPS) (pH 6.9), (iv) 0.1 M MOPS (pH 7.2), (v) 0.1 M phosphate (K^+^) (pH 7.4), or (vi) 0.1 M Tris/HCl (pH 8.0). The same solutions supplemented with 0.3 mM ZnSO_4_ were also assayed. Reaction mixtures were prepared directly on the microplate’s wells and had a volume of 100 μL. The NDM-1 MBL and the NMC-A class A carbapenemases were tested, and results were compared with those of control wells.

The effects of NDM-1 and NMC-A carbapenemases on 5 mg/mL of imipenem (imipenem hydrate, ≥98%; Cayman Chemicals) solution containing 16 mM NaHCO_3_ and 0.15 mM ZnSO_4_ as well as on 5 mg/mL imipenem-5 mg/mL cilastatin in 0.15 mM ZnSO_4_ prepared from the brand name Primaxin formulation (Merck, Sharp & Dohme Corp.) were also examined.

### Analysis of bacterial suspensions with the imipenem decomposition method.

Dense cell suspensions were prepared with the addition of two full 10-μL plastic inoculation loops (Sarstedt, Germany) of bacteria grown on tryptone soya agar (TSA) (Oxoid-Thermo Scientific, UK), supplemented with 0.3 mM ZnSO_4_, into 400 μL of H_2_O. For each strain, 50 μL of this suspension was added into four wells of a 96-well microplate (polystyrene flat bottom clear wells; Greiner, Germany). Fifty microliters of the imipenem/cilastatin-ZnSO_4_ stock solution were added in two of the above wells, while in the remaining two, introduced for absorbance correction, the same volume of a 0.3 mM ZnSO_4_ solution was added (control 1). Wells containing the imipenem/cilastatin- ZnSO_4_ (control 2) and ZnSO_4_ solutions (control 3) diluted 1:1 with H_2_O were also included as controls. The plates were incubated at 37°C, and the absorbance was measured using a Dynex MRX microplate reader at various time points. The absorbance of the wells containing the mixtures of bacterial suspensions with imipenem/cilastatin were corrected by subtracting that of control 1 and control 2 (control 3 corrected) wells. Each experiment was performed in triplicate. Estimation of a threshold of absorbance increase in order to characterize a strain as a carbapenemase producer was carried out through receiver operating characteristic (ROC) analysis with Prism v. 8.0. Absorbance changes documented in experiments of carbapenemase-negative strains were grouped in the “control column,” and those of positive strains in the “patients” column. An absorbance increase greater than 0.045 yielded 98% sensitivity (95% confidence interval [CI], 89.5 to 99.9%) and 100% specificity (95% CI, 98.1 to 100.0%). Hence, the threshold was set at 0.05 absorbance increase.

The effect of metal chelation on color development was assessed by the addition of EDTA (0.5 M, pH 8) in the bottom of the wells before the various reaction mixture components and by preparing the bacterial suspensions in EDTA-containing solutions. The concentrations of EDTA included in the reaction during preliminary experiments were 10 and 15 mM, with the former being selected as optimum. In these experiments, bacteria grown on TSA without zinc supplementation were also tested.

### Comparisons with the Carba NP technique.

Direct colorimetry was compared with the commercial pH indicator colorimetric technique Rapidec Carba NP (bioMérieux, France). For these comparisons, we included strains expected to cause sensitivity and specificity issues in CPE detection techniques. Bacteria were grown on TSA containing 0.3 mM ZnSO_4_ at 37°C for 16 h, and the assay was performed and interpreted according to the manufacturer’s instructions.
